# Case Report: Giant congenital transmesenteric hernia in a 6-month-old infant: a case of delayed recognition and critical diagnostic lessons

**DOI:** 10.3389/fped.2025.1638744

**Published:** 2025-11-07

**Authors:** Xinggui Fang, Benquan Wang, Biao Yang, Qun Gao

**Affiliations:** 1 Department of Pediatric Surgery, The First People’s Hospital of Wuhu, Anhui, China; 2 Department of Gastrointestinal Surgery, The First People’s Hospital of Wuhu, Anhui, China; 3 Department of Pediatric Surgery, Suzhou Hospital, Affiliated to Anhui Medical University, Suzhou, China

**Keywords:** congenital transmesenteric hernia, internal hernia, acute abdominal pain, intestinal obstruction, infant

## Abstract

Congenital transmesenteric hernia, a rare internal hernia, is notoriously challenging to diagnose preoperatively due to nonspecific symptoms and inconclusive routine evaluations. In infants, early manifestations such as vomiting, irritability, and diarrhea often mimic acute gastroenteritis, leading to delayed recognition. Without timely intervention, progression to intestinal strangulation, necrosis, and shock significantly escalates treatment complexity and mortality risk. We report a case of a 6-month-old female infant with approximately 131 cm of necrotic small bowel secondary to a giant transmesenteric hernia. This case illustrates the clinical trajectory, diagnostic pitfalls, and surgical management, highlighting the imperative for early suspicion and intervention. Critical analysis of this case underscores that prompt surgical correction is pivotal to mitigate morbidity and mortality in such scenarios. Enhanced clinician awareness of this condition and its subtle early signs could improve outcomes in pediatric patients.

## Introduction

Congenital transmesenteric hernia is a type of internal hernia in which the intestinal tract herniates through a congenital defect in the mesentery (1, 2). This condition represents one of the rare causes of acute intestinal obstruction, accounting for approximately 0.5%–3% of all internal hernia etiologies (3). It predominantly affects children and is marked by rapid onset and progression, with a high risk of intestinal strangulation, gangrene, and septic shock occurring within a short timeframe (4, 5). Given the challenges associated with preoperative diagnosis and its high mortality rate, this disease has garnered considerable clinical attention.

Individuals across different age groups exhibit notable differences in clinical presentations. Specifically, infants under one year of age, who have not yet developed verbal communication skills, are unable to clearly describe their symptoms and often display non-cooperative behavior during medical examinations. Consequently, these factors can lead to delayed diagnoses or clinical misjudgments. The pathological progression of an internal hernia through a mesenteric defect becomes life-threatening once complicated by strangulating intestinal obstruction and transmural bowel necrosis. This critical condition constitutes a surgical emergency requiring immediate intervention to prevent imminent risk of fatal complications (6–8). This article reports the clinical course of a 6-month-old female infant in whom initial evaluation revealed inconclusive symptomatology, equivocal physical findings, and inconclusive diagnostic imaging. This diagnostic uncertainty delayed definitive categorization of the pathology. Notably, the patient manifested acute clinical deterioration during the 12-h pre-surgical observation window. Critical analysis of serial diagnostic evaluations coupled with targeted therapeutic interventions allowed preoperative confirmation of small bowel necrosis, later corroborated by intraoperative findings. Surgical exploration ultimately attributed the etiopathogenesis of intestinal strangulation and transmural necrosis to an extensive congenital mesenteric-defect hernia.

This case presents an exceptionally rare case of a giant transmesenteric hernia encountered in clinical practice. The uniqueness of this case lies in the patient's young age, extensive intestinal necrosis, and multiple complications. These factors significantly complicated the preoperative diagnostic process and underscored the essential role of multidisciplinary collaboration in managing such complex and critical cases.

## Case presentation

This case involves a 6-month-old female infant presenting to our pediatric emergency department at 23:10 h on September 19, 2024, accompanied by legal guardians. Clinical history revealed a 12-h preadmission period marked by six discrete episodes of non-bilious emesis accompanied by three watery stools, notably lacking hematochezia or melena. Progressive symptom escalation manifested as febrile onset (core temperature 38.9 °C) at the sixth hour post-symptom initiation. Initial management at a primary care facility included intravenous fluid resuscitation and Ceftriaxone Sodium (50 mg/kg) following provisional diagnosis of acute gastroenteritis. Although standard antimicrobial therapy was administered, clinical deterioration ensued with emergence of progressive tachypnea (respiratory rate 32/min), altered mental status, and recurrent convulsions demonstrating generalized tonic-clonic characteristics. The patient experienced paroxysmal convulsions during febrile episodes, with each seizure lasting ≤1 min. A sedative (phenobarbital sodium 4 mg/kg intramuscularly) was administered during the acute phase. Notably, the patient had a documented history of febrile seizures. Upon admission, the patient presented with lethargy and severe dehydration. Initial vital signs indicated fever (38.3 °C), marked tachycardia (190 bpm), hypotension (75/46 mmHg), and tachypnea (32 breaths/min). Abdominal examination revealed a soft, non-distended abdomen with no tenderness, muscle rigidity, or palpable masses. Hyperactive bowel sounds (8/min) were observed, and the digital rectal examination yielded no abnormalities. This therapeutic impasse necessitated emergent transfer to our tertiary care center for advanced diagnostic workup and critical care management.

Physical examination revealed fever (38.5 °C), tachycardia (200 bpm; exceeding 99th percentile for age), tachypnea (34 breaths/min), and hypotension (70/43 mmHg,). Clinical findings included lethargy (Blantyre Coma Scale 3/5), moderate dehydration (5% weight loss), and respiratory distress with fine bibasilar crackles. Abdominal assessment demonstrated hyperactive bowel sounds (8/min) without peritoneal signs. Laboratory analysis showed leukocytosis (WBC 19.8 × 10⁹/L, 90th percentile), elevated C-reactive protein (CRP) (11.8 mg/L; normal: <5), normocytic anemia (HGB: 93 g/L; normal: 110–140 g/L, and critical hyponatremia (Na^+^: 117 mmol/L; normal: 135–145 mmol/L). The routine fecal examination results showed weakly positive reactions for red blood cells and white blood cells. Arterial blood gas analysis performed prior to intervention revealed metabolic acidosis with respiratory compensation: pH: 7.31 (normal: 7.35–7.45), PaO_2_: 96 mmHg, PaCO_2_: 20.4 mmHg (normal: 35–45 mmHg), HCO_3_^−^: 12.6 mmol/L (normal: 22–27 mmol/L), lactate 4.4 mmol/L (normal: 0.5–1.6 mmol/L). Imaging studies: Chest x-ray demonstrated bilateral lower lobe consolidations with air bronchograms. Abdominal upright radiograph revealed absence of pneumoperitoneum or pathognomonic air-fluid levels. Abdominal ultrasound demonstrated unremarkable findings with preserved bowel wall architecture, normal peristalsis patterns, and absence of free intraperitoneal fluid or air. The preliminary diagnosis included bilateral pneumonia, severe hyponatremia, and suspected infectious enterocolitis. Subsequently, the patient was admitted to the pediatric ward and received comprehensive treatment, including nil per os (NPO), Imipenem/Cilastatin (20 mg/kg), fluid resuscitation, and oxygen supplementation.

**Figure 1:**
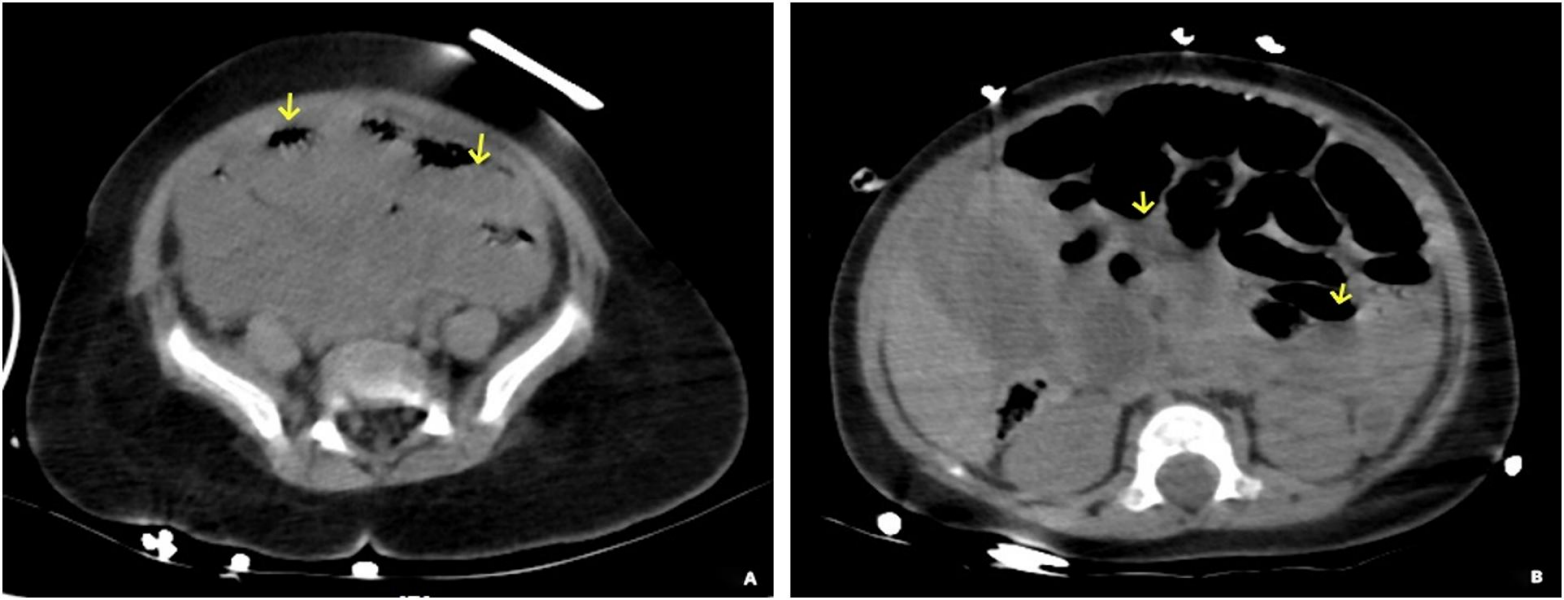
Abdominal CT non-contrast imaging demonstrates dilatation of the small intestine accompanied by a visible air-fluid level (the arrows in panels **A** and **B**), consistent with the presence of small bowel obstruction.

Following 6 h of therapeutic intervention in the pediatric ward, the patient's clinical status exhibited no substantial improvement. Persistent pyrexia, recurrent convulsive episodes, and tachypnea were documented. Despite supplemental oxygen therapy via nasal cannula, oxygen saturation deteriorated from 100% on admission to below 80%, signifying profound respiratory compromise. Auscultation revealed diminished bilateral vesicular breath sounds accompanied by expiratory wheezing. Abdominal examination demonstrated mild distension without peritoneal signs. Computed tomography (CT) of the thorax demonstrated extensive bilateral consolidations with interstitial infiltrates. Abdominal CT imaging demonstrated mild dilation of the small intestinal loops without evident gas-fluid levels (Figure 1). Additionally, no free fluid was identified within the abdominal or pelvic cavities. Serial laboratory investigations revealed leukocytosis (WBC: 9.2 × 10⁹/L), elevated inflammatory markers (CRP: 46.1 mg/L), and moderate anemia (HGB: 65 g/L). Progressive clinical deterioration manifested by multiorgan dysfunction syndrome (MODS) necessitated emergent transfer to the pediatric intensive care unit (PICU) for invasive mechanical ventilation and vasopressor support.

**Figure 2:**
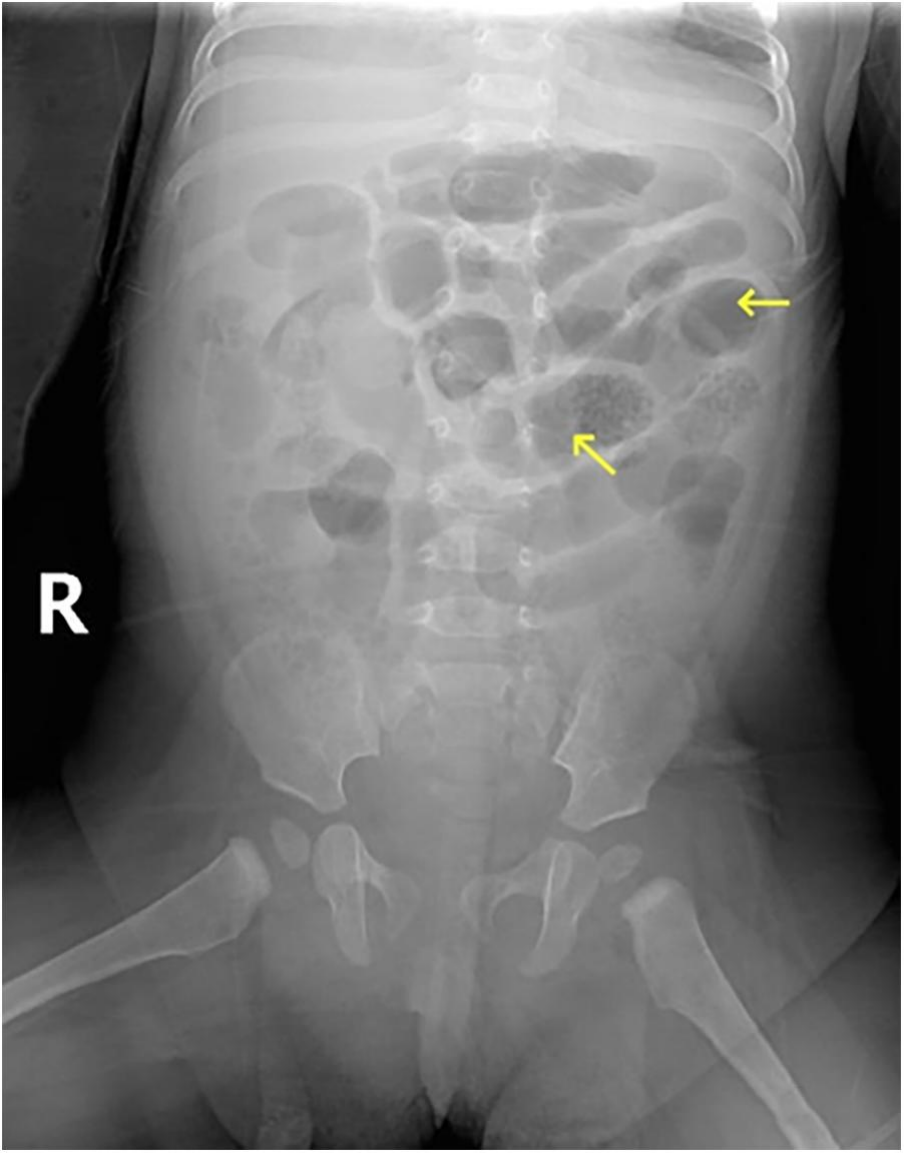
Bedside abdominal supine view demonstrating small bowel dilatation and gas accumulation, with no evidence of free intraperitoneal air.

After 4 h of the pediatric intensive care unit (PICU) management, the patient demonstrated sustained and progressive clinical deterioration characterized by refractory hypotension (mean arterial pressure <35 mmHg), profound pallor, cold extremities with mottling, marked abdominal distension, and generalized muscular rigidity. Serial laboratory investigations revealed leukopenia with severe neutropenia (WBC: 2.1 × 10⁹/L, absolute neutrophil count <0.5 × 10⁹/L), markedly elevated CRP (94.6 mg/L), and critical anemia (HGB: 51 g/L). A bedside abdominal supine radiograph revealed small bowel dilatation and intraluminal gas accumulation (Figure 2). Point-of-care ultrasonography revealed intramural gas patterns within the small intestinal wall, highly indicative of transmural necrosis, as well as minimal free fluid in both the abdominal and pelvic cavities. Ultrasound-guided diagnostic paracentesis yielded hemorrhagic transudate. These findings confirmed septic shock secondary to transmural intestinal necrosis, prompting immediate surgical consultation.

**Figure 3:**
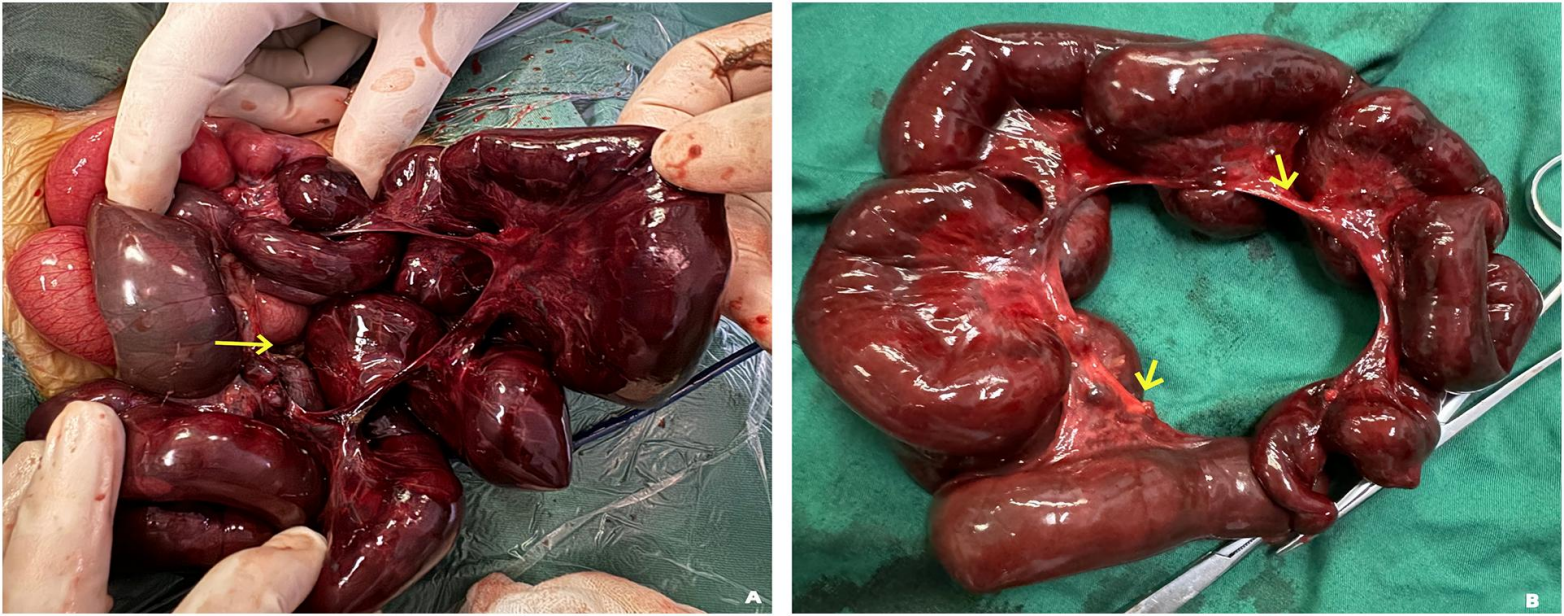
**(A)** intraoperative findings demonstrate a congenital mesenteric defect measuring approximately 18 cm × 15 cm, accompanied by extensive incarceration and necrosis of the small bowel (arrows). **(B)** A segment of necrotic small bowel, measuring approximately 131 cm in length, was surgically resected. The smooth, rounded morphology of the mesenteric margins is consistent with the characteristic appearance of a mesenteric hiatus defect (arrows).

Concurrently with ongoing shock management and blood product resuscitation, our pediatric surgery team performed an urgent exploratory laparotomy. Intraoperative findings included a 131 cm segment of gangrenous small bowel herniating through an 18 cm × 15 cm congenital mesenteric defect. The gangrenous bowel extended from 100 cm distal to the ligament of Treitz to 80 cm proximal to the ileocecal valve, demonstrating complete vascular compromise. The resected necrotic tissue encompassed the entire necrotic portion of the small intestine along with the congenital mesenteric defect (Figures 3A,B). Following the successful completion of end-to-end anastomosis between viable intestinal segments, the residual mesenteric defect near the resection site was repaired in a systematic and anatomically precise manner using interrupted 3-0 polypropylene sutures.

Systematic peritoneal lavage was then performed with 2,000 ml of warmed (37 °C) isotonic saline under low-pressure irrigation. The surgical procedure was completed in 65 min, during which the patient's hemodynamic parameters remained stable throughout. Histopathological evaluation of the resected intestinal segment demonstrated ischemic necrosis involving the small bowel.

The patient achieved uncomplicated recovery with discharge on postoperative day 10. Standardized predischarge evaluation verified tolerance of regular diet with absence of diarrheal events. Serial post-discharge monitoring through 6-month follow-up demonstrated age-appropriate growth parameters and normal developmental milestones, without clinical evidence of nutritional deficits or gastrointestinal complications including diarrhea.

## Discussion

Mesenteric hernia-induced acute intestinal obstruction remains a rare clinical entity in pediatric surgery, particularly among infants under 12 months constituting a surgical emergency. Diagnostic uncertainties frequently precipitate progressive bowel compromise, with delayed recognition (>24 h) culminating in catastrophic outcomes (9). Early surgical intervention emerges as the gold-standard approach, demonstrating 92% survival rates when implemented within 6 h of presentation. Contemporary registry analyses indicate mesenteric defects underlie 0.5%–3% of pediatric mechanical obstructions, with peak incidence during neonatal transition phases. Despite technological advances, 8.7% of cases progress to transmural necrosis, translating to 50% mortality rates in under-3-month cohorts, particularly within the first 72 h of symptom onset (10, 11). Diagnostic precision remains paramount in neonates, requiring multimodal imaging protocols combining contrast-enhanced ultrasonography and low-dose CT enterography (12). Current embryological evidence from fetal autopsy series supports two distinct pathogenic mechanisms: (1) persistent mesenteric vascular insufficiency causing developmental defect formation (SOMIT Trial 2021 findings), and (2) developmental malformation of peritoneal fusion planes as documented in Langman's Medical Embryology (14th ed, Chapter 12).

Transmesenteric hernia exhibits a non-specific clinical presentation with a notable symptom-sign dissociation during its initial symptomatic phase, characterized by a triad of bilious vomiting (occurring in 82% of cases), colicky abdominal pain, and loose stools. It is crucial to recognize that congenital transmesenteric hernia represents a persistent anatomical anomaly; symptomatic onset is typically precipitated by secondary complications such as vascular compromise or intestinal entrapment. During this early symptomatic stage, abdominal examination reveals normal bowel sounds in 67% of patients (13). Progression to closed-loop obstruction triggers rapid hemodynamic compromise, with 30% developing hypovolemic shock within 4–6 h of onset secondary to transmural necrosis. Diagnostic challenges escalate in infants <6 months due to: 1) preverbal status precludes pain localization, 2) 43% incomplete history from caregivers, 3) limited physical exam reliability, and 4) absence of specific imaging features, collectively contributing to 22% misdiagnosis rate in neonatal intensive care units (NICUs).

Preoperative diagnostic accuracy for mesenteric defects remains limited, with 83% of cases confirmed during emergent laparotomy for small bowel obstruction (14). Pathophysiological divergence stems from mechanical dynamics—smaller defects induce axial torsion compromising arcades, whereas larger defects permit mesenteric mobility reducing vascular compromise (15). Chronic presentations (23% of cases) feature postprandial periumbilical pain and cyclic vomiting, often misdiagnosed as functional gastrointestinal disorders. Complete strangulation precipitates pan-enteric necrosis within 6-h critical window, escalating to distributive shock in 68% of neonates (16, 17). This case report describes an infant presenting with a large congenital transmesenteric hernia The patient's clinical condition deteriorated significantly within 12 h, as the initial nonspecific symptoms and ambiguous imaging findings greatly impaired diagnostic accuracy. The concurrent presence of a severe pulmonary infection further complicated clinical management and decision-making. Collectively, these factors indicate that this case represents an exceptionally rare and challenging scenario in clinical practice. The insidious progression of the condition, combined with multifactorial confounding factors, highlights diagnostic challenges inherent to the pathology rather than clinical oversight. In the absence of definitive radiological evidence, surgical decision-making becomes particularly challenging.

A comprehensive literature (4, 5, 6, 18) review of similar clinical cases demonstrates a predominance of reports focusing on adult and school-aged populations, with relatively few descriptions involving infants and young children. In documented cases of internal herniation through mesenteric defects, the anatomical breaches are typically small (≤5 cm) and clinically associated with acute intestinal obstruction, allowing for relatively straightforward surgical decision-making. However, this case presented with exceptional diagnostic and therapeutic complexity, attributable to the following distinctive clinical features: Firstly, the patient was an infant presenting in a critically ill condition, already exhibiting early signs of septic shock upon initial evaluation. The combination of atypical clinical manifestations and limitations in diagnostic imaging delayed definitive diagnosis, leading to rapid clinical deterioration and progression to multi-organ dysfunction syndrome (MODS) within a short time frame. These cascading complications significantly complicated the diagnostic process and delayed the initiation of optimal surgical intervention. Secondly, the patient was found to have an unusually large mesenteric defect measuring 18 cm × 15 cm, which is extremely rare in the current surgical literature. Interestingly, although such large defects may reduce the risk of intestinal strangulation due to the absence of significant luminal narrowing, they carry a high potential for extensive bowel necrosis once obstruction occurs, thereby significantly increasing the complexity of the clinical course and management.

Heightened clinical vigilance toward rare diseases and standardized differential diagnostic algorithms are imperative for risk mitigation. Despite its low recognition among clinicians, this form of internal herniation carries significant mortality risks when left untreated (19). This clinical case highlights how congenital transmesenteric hernia, which presents with nonspecific early symptoms, frequently leads to delayed or incorrect initial diagnoses. Consequently, we advocate for enhanced disease-specific education and systematic implementation of multidisciplinary care pathways to reduce diagnostic inaccuracies, ultimately improving therapeutic efficacy and survival outcomes.

## Conclusion

This case highlights critical shortcomings in clinical practice, including delayed recognition of warning signs and postponed multidisciplinary team involvement, which can lead to delayed diagnosis. Key lessons emphasize the prognostic significance of elevated lactate levels and the diagnostic value of multimodal imaging for early identification. Implementing an improved protocol may reduce the risk of intestinal necrosis and improve patient outcomes. This case report provides clinically relevant insights that support the timely and proactive surgical management of patients with similar conditions.

## Data Availability

The original contributions presented in the study are included in the article/Supplementary Material, further inquiries can be directed to the corresponding author.
